# Anemic retinopathy as the presenting symptom of a mediastinal germ cell tumor

**DOI:** 10.1016/j.ajoc.2025.102374

**Published:** 2025-06-27

**Authors:** Christian Tallo, Paul Farris, Edmund Farris

**Affiliations:** a University of Connecticut School of Medicine, Farmington, CT, USA; b Quinnipiac University Frank H. Netter MD School of Medicine, North Haven, CT, USA; c UConn Health, Department of Ophthalmology, Farmington, CT, USA

**Keywords:** Anemic retinopathy, Retinopathy, Anemia, Germ cell tumor, Mediastinal mass

## Abstract

**Purpose:**

To describe a rare case of anemic retinopathy as the initial presenting symptom of a mediastinal germ cell tumor.

**Observations:**

A 27-year-old male with no prior medical history presented with a one-day history of central vision loss in his left eye. Funduscopic evaluation showed a central macular hemorrhage in the left eye and bilateral nerve fiber layer hemorrhages and blot hemorrhages. Fluorescein angiography showed multiple hemorrhages without vascular occlusion or neovascularization, features indicative of anemic retinopathy. Laboratory evaluation demonstrated severe anemia (hemoglobin 6.7 g/dL), with an otherwise unremarkable metabolic and coagulation profile. Given these findings, an underlying hematologic or oncologic disorder was suspected, prompting further systemic evaluation. Imaging revealed a large anterior mediastinal mass, and biopsy confirmed a nonseminomatous germ cell tumor. The patient underwent chemotherapy with cisplatin, etoposide, and bleomycin, later transitioning to VIP (ifosfamide, etoposide, and cisplatin) due to suspected pulmonary toxicity. Patient responded well to treatment; his anemia gradually improved, and alpha-fetoprotein levels significantly declined. Following treatment, the patient reported no ongoing visual disturbances.

**Conclusions and importance:**

Anemic retinopathy can be the initial presentation of an underlying malignancy. Recognizing ophthalmologic findings as potential indicators of systemic disease is critical for early diagnosis and timely intervention in cases of occult malignancies.

## 1 Introduction

Ophthalmologic findings are a rare initial presentation of systemic malignancies. Retinopathy of anemia, a condition characterized by retinal hemorrhages and vascular changes due to severe anemia, is often asymptomatic.^1^ When symptomatic, it typically manifests as transient vision loss, with the severity of retinal involvement correlating with the degree of anemia.^2^

**Fig. 1:**
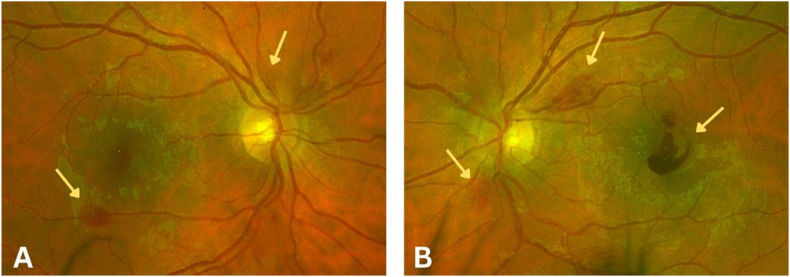
Fundus photography. (A) Nerve fiber layer and blot hemorrhages in the right eye. (B) Large macular hemorrhage with associated nerve fiber layer and blot hemorrhages in the left eye.

**Fig. 2:**
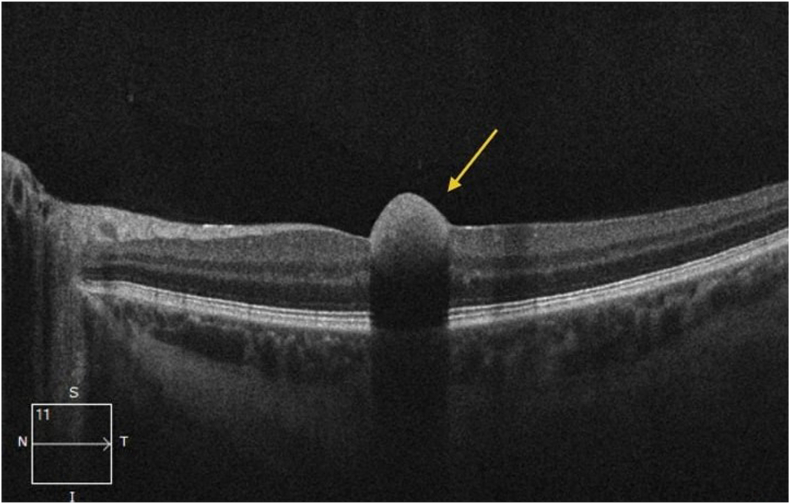
Optical coherence tomography of the macula in the left eye demonstrating a hyperreflective, dome-shaped retinal lesion consistent with macular hemorrhage.

**Fig. 3:**
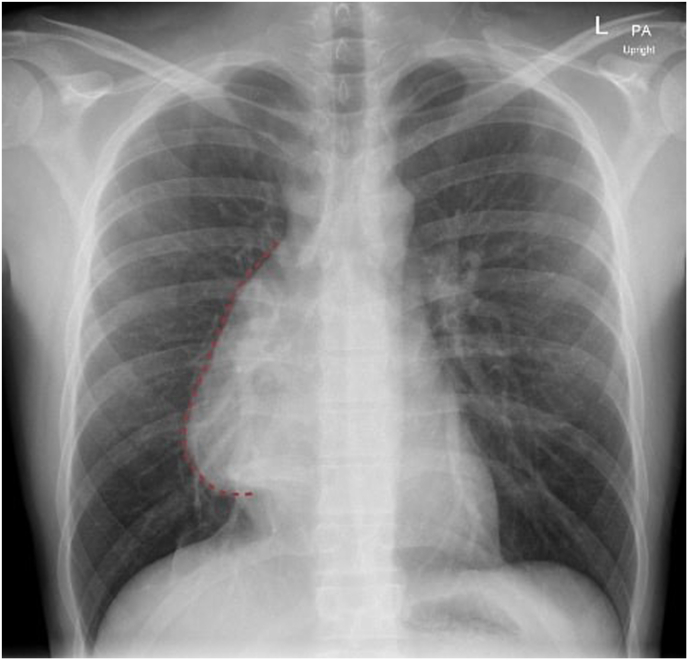
Chest x-ray demonstrating a new prominent density along the right heart border, suspicious of an anterior mediastinal mass.

**Fig. 4:**
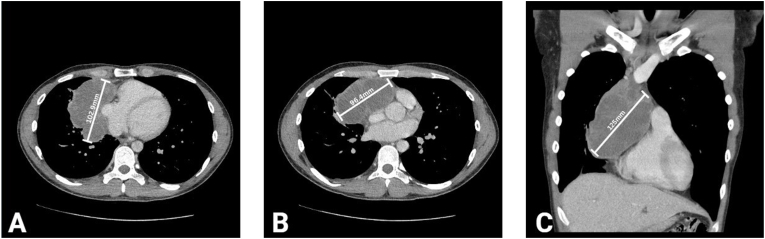
CT chest with IV contrast demonstrating a large anterior mediastinal mass with concomitant superior mediastinal lymphadenopathy. (A) Axial cut of the mediastinal mass with a largest anteroposterior diameter of 102.9mm. (B) Axial cut of the mediastinal mass with largest transverse diameter of 96.4mm. (C) Coronal cut with a peak diameter of 125mm.

Germ cell tumors (GCTs) most commonly originate in the gonads; however, approximately 5 % of cases arise from extragonadal sites due to aberrant migration of germ cells during fetal development.^3^ The mediastinum is the most frequent location for extragonadal germ cell tumors (EGCTs) in males, particularly between the ages of 20 and 35, and these tumors are typically associated with elevated levels of beta-hCG and/or alpha-fetoprotein 4. Their clinical presentation is often dictated by tumor mass effect, leading to symptoms such as dyspnea, vascular compression, Horner's syndrome, or arrhythmias.^5^

It is rarer for mediastinal EGCTs or metastatic testicular GCTs to initially manifest with ocular symptoms, such as vision loss, from their more common presentation from an underlying mass effect.^5^ Here, we describe a rare case of a young male who presented to an ophthalmology clinic with acute vision loss due to a macular hemorrhage and nerve fiber layer hemorrhages related to anemic retinopathy, ultimately leading to the diagnosis of a mediastinal EGCT. This case underscores the importance of recognizing ophthalmologic findings as potential harbingers of systemic disease, particularly in patients without a prior medical history.

## 2 Case presentation

A 27-year-old male with no significant past medical history presented with a one-day history of central vision loss in the left eye. Ophthalmologic examination revealed visual acuity (Snellen) of 20/20 OD and 20/60 OS. The anterior segment exam was otherwise unremarkable. Fundus examination revealed a central macular hemorrhage in the left eye, presumed to be the cause of vision loss, along with additional nerve fiber layer hemorrhages surrounding the optic nerve and near the macula in both eyes (Fig. 1). Fluorescein angiography demonstrated multiple bilateral hemorrhages without evidence of vasculitis, vascular occlusion, or neovascularization. Fundus photography confirmed these hemorrhages, while optical coherence tomography (OCT) showed evidence of a significant macular hemorrhage in the left eye (Fig. 2). Because of these findings there was concern for a systemic process and further testing was initiated.

Laboratory evaluation revealed severe anemia, with a hemoglobin level of 6.7 g/dL (normal range: 13–18 g/dL) and a hematocrit of 20.2 % (normal range: 40–52 %). A complete blood count (CBC) demonstrated an elevated neutrophil percentage of 75.5 % (normal range: 40–70 %) and decreased lymphocytes at 14.1 % (normal range: 20–50 %), markedly different from a previously normal CBC recorded two years prior. Hemoglobin A1c was within normal limits. Given these findings, the patient was diagnosed with anemic retinopathy, suspected to be secondary to an underlying oncologic or hematologic disorder, and was referred to the emergency department for urgent evaluation and possible transfusion.

Upon admission, patient was asymptomatic despite significant physical exam findings of moderate skin pallor and fever. Laboratory findings included an elevated ferritin level of 1603 ng/mL (normal range: 16–336 ng/mL), lactate dehydrogenase of 270 U/L (normal range: 125–220 U/L), and an INR of 1.3 (normal range: 0.9–1.1). Liver function tests and bilirubin levels were within normal limits, with no evidence of metabolic derangements. A chest X-ray revealed a new density along the right heart border, suspicious for mediastinal mass, prompting further imaging (Fig. 3). Computed tomography of the chest identified a large anterior mediastinal mass (9.6 × 10.3 × 12.5 cm) with associated superior mediastinal lymphadenopathy, raising suspicion for lymphoma, germ cell tumor, or primary thymic neoplasm (Fig. 4). Hematology-oncology consultation was obtained, and laboratory analysis demonstrated an elevated alpha-fetoprotein (AFP) of 1459 ng/mL (normal range: 0–10 ng/mL) with a normal beta-hCG level of 6 IU/L. Core biopsy confirmed a diagnosis of germ cell neoplasia.

The patient received a transfusion of two units of packed red blood cells, resulting in an increase in hemoglobin to 9.2 g/dL. However, subsequent CBCs showed persistent anemia and thrombocytopenia, with a platelet count of 105 × 10^3^/μL (normal range: 150–440 × 10^3^/μL). A bone marrow aspirate was performed to investigate potential marrow involvement of germ cell neoplasia, but no morphologic abnormalities were identified; however, genetic analysis revealed TP53 and CBL mutations.

He began receiving oncological treatment and follow-ups through an outside hospital. His initial chemotherapy consisted of bleomycin, etoposide, and cisplatin. However, due to suspected pulmonary toxicity from bleomycin, his regimen was transitioned to VIP (ifosfamide, etoposide, and cisplatin). He remained thrombocytopenic and anemic throughout chemotherapy and routinely required platelet transfusions with the expected hematologic response, while his anemia improved gradually and has remained stable. While his thrombocytopenia and anemia were not further evaluated, it was closely monitored due to its known synchronous associations with germ cell tumors.

Before completing his fourth chemotherapy cycle, AFP levels had significantly improved to 14.6 ng/mL, and beta-hCG remained within normal limits. The patient declined further formal ophthalmologic follow-up but continued care with his hematology team. During a telephone follow up, he denied any visual disturbances, including blurriness or visual auras. Patient unfortunately succumbed to his disease shortly after this encounter.

## 3 Discussion

Anemic retinopathy results from retinal hypoxia causing an infarct in the nerve fiber layer. Hypoxia can also lead to vascular dilatation, and increased transmural pressure causing microtrauma to the vessel walls.^1^ The central retinal artery's deep capillary and superficial plexuses, which supply blood to the inner retinal layers, are the most vulnerable to hypoxic changes.^6^ Coexisting thrombocytopenia also leads to defective coagulation and hemorrhage.^1^ Patients are usually asymptomatic, but the physical signs include conjunctival pallor, retinal hemorrhage, cotton wool spots, venous tortuosity, and Roth spots.^6^ The incidence of retinopathy in anemic patients is 28 % when the hemoglobin is below 8 g/dL, and is 38 % when there is coexisting thrombocytopenia.^7^ The severity of the retinopathy increases as hemoglobin decreases, and symptoms are noticeably worse once the hemoglobin levels drop below 6 g/dL.^1^ Retinal changes can be the first sign of blood disorders.^8^

The findings of blot hemorrhages located inferior to the macula and superior and inferior to the optic nerve of the asymptomatic eye initiated concern and necessitated bloodwork for further investigation, leading to the diagnosis of malignancy. Our patient exhibited an initial Hgb level of 6.7g/dl without corresponding thrombocytopenia. Severe anemic retinopathy has not previously been reported as the initial presentation for germ cell tumors.^9^ The differential diagnoses for bilateral retinal hemorrhages include hypertensive retinopathy, diabetic retinopathy, retinal vein occlusion, blood dyscrasias, and trauma. In most cases, however, patients are symptomatic in both eyes. Even if not, the absence of cotton wool spots or venous dilation in our patient argued against vein occlusion or hypertensive crisis, while the absence of microaneurysms and exudates made a diabetic etiology unlikely. Furthermore, these were further ruled out with unremarkable vital signs and A1c, causing anemia to emerge as the most plausible systemic driver and was confirmed by laboratory studies.

It is also important to understand the context behind the location of the hemorrhage. Some locations of subretinal hemorrhages produce the monocular central vision loss that prompted our patient's presentation.^10^ Review of the color fundus photographs and, in particular, the OCT revealed a dome-shaped, hyper-reflective collection that separated the internal limiting membrane from the underlying retina, findings diagnostic of a sub-internal limiting membrane (sub-ILM) hemorrhage. Blood in this compartment can cast a dense shadow over the fovea, readily explaining the patient's decrease in visual acuity. The intact posterior hyaloid membrane and absence of overlying hyper-reflective material on OCT effectively rule out vitreous or sub-hyaloid hemorrhage as alternative sources, other variants that may decrease central vision.^10^ Sub-ILM hemorrhages can follow abrupt rises in intrathoracic pressure.^10^ It is plausible that an unrecognized Valsalva-type event, superimposed on the vessel fragility caused by profound anemia, precipitated this macular bleed. Therefore, also recognizing the anatomic level of the hemorrhage can be crucial to precipitate when to evaluate for anemia and underlying causes.

Different mechanisms exist as to how germ cell tumors can cause anemia. Cancer causes cytokine release from the interactions between the immune system and tumor cells. This cytokine release can cause shortened lifespan of red blood cells, the suppression of erythroid progenitor cells, and inadequate erythropoietin production.^11^ TNF-alpha is a cytokine that inhibits hemoglobin proliferation. It indirectly inhibits the proliferation of erythroid progenitor cells by triggering NF-kB and GATA-2, which suppresses erythropoietin production.^9^ Cancer can also infiltrate the bone marrow and decrease red blood cell production.

Germ cell tumors arise from the reproductive cells of the testes or ovaries, along the track of migration of primordial germ cells. Presentations of mediastinal germ cell tumors can be very nondescript, and symptoms include cough, dyspnea, fever, night sweats, weight loss, and chest pain. SVC compression can cause facial plethora and prominent neck veins, and bronchus compression can cause post-obstructive pneumonia and hemoptysis. These findings are usually asymptomatic until they are advanced and large.^12^ Our patient had none of these presenting symptoms, and the workup that led to the tumor diagnosis came only after an abnormal CBC, ordered because of the initial presentation of nerve fiber hemorrhages including the affected as well as the asymptomatic eye.

Only 1–5 % of germ cell tumors are extragonadal, and most are found in the mediastinum, retroperitoneum, pineal gland, or suprasellar area.^12^ 54 % of extragonadal germ cell tumors are mediastinal.^13^ 3–10 % of mediastinal tumors are germ cell tumors.^11^ They mostly affect children and young adults. AFP, LDH, and b-HCG are tumor markers that are often elevated.^12^ Not all of the tumor markers must be elevated, and our patient had a normal b-HCG but elevated AFP.

Chemotherapy and surgery are used as treatment of these tumors. The standard treatment is multi-agent chemotherapy followed by salvage surgery to resect residual tumor. Cisplatin with bleomycin and etoposide was used in this patient, but ifosfamide can be used instead of bleomycin in patients with lung issues, as pulmonary toxicity is a potential side effect of bleomycin 12. 4 cycles of bleomycin, etoposide, and cisplatin is the standard.^14^ Surgical resection is recommended if serum tumor markers remain elevated after chemotherapy or if the residual mass is greater than 3 cm. AFP and b-HCG should also be tracked throughout the treatment process.^12^ Our patient's AFP declined with treatment.

Prior to cisplatin-based chemotherapy, cure rates were below 10 %.^14^ Now there is a 5 year survival rate of 48–64 % for germ cell tumors that meet poor prognosis criteria, which includes one of the following: mediastinal primary tumors, nonpulmonary visceral metastases, AFP levels greater than 10,000 ng/mL, or b-hCG levels greater than 50,000 IU/L.^15^

Chemotherapy regimens may have complications. 5-HT3 antagonists are helpful to decrease nausea and vomiting. Bleomycin can cause neutropenic fever and severe thrombocytopenia. This can require the use of hematopoietic growth factors. If pulmonary toxicity is suspected, bleomycin should be discontinued if imaging shows changes in the lung or if DLCO decreases by 30 %. Cisplatin can cause nephrotoxicity, and it is cumulative. Hypomagnesemia is common and may require supplementation. Cisplatin can also cause auditory toxicity with reduced hearing of high-tone sounds. Mediastinal radiotherapy has also been linked to advanced coronary artery disease.^14^ Our patient discontinued use of bleomycin for suspected pulmonary toxicity.

Our case shows that germ cell tumors and other solid malignancies must be considered with an initial presentation of anemic retinopathy. Our patient's only presenting symptom was one day of central visual acuity loss in the left eye, and nerve fiber layer hemorrhages were seen without any other significant findings. Our patient declined further ophthalmic follow-up due to focusing on intense mediastinal tumor treatment.

## 4 Conclusion

This case highlights anemic retinopathy as a rare presenting symptom of a mediastinal germ cell tumor. While anemia is a known consequence of malignancy, its ocular manifestations are often overlooked. Our patient's diagnosis was initiated by ophthalmologic findings, emphasizing the critical role of retinal examination in detecting systemic disease, and the need for clinicians to consider malignancy in patients with unexplained anemic retinopathy.

### 4.1 Patient consent

Written consent to publish this case has not been obtained. This report does not contain any personal identifying information.

## CRediT authorship contribution statement

**Christian Tallo:** Writing – review & editing, Writing – original draft, Formal analysis, Data curation. **Paul Farris:** Writing – review & editing, Writing – original draft, Formal analysis, Data curation. **Edmund Farris:** Writing – review & editing, Supervision, Project administration, Methodology, Conceptualization.

## Authorship

All authors attest that they meet the current ICMJE criteria for Authorship.

## Funding

No funding or grant support.

## Declaration of competing interest

The authors declare that they have no known competing financial interests or personal relationships that could have appeared to influence the work reported in this paper.
